# Trisomy 26 in a Holstein calf with disorders of sex development

**DOI:** 10.1111/age.13489

**Published:** 2024-11-03

**Authors:** Markus Freick, Joana G. P. Jacinto, Irene M. Häfliger, Jim Weber, Holger Behn, Ruben Schreiter, Cord Drögemüller

**Affiliations:** ^1^ Institute of Agricultural and Nutritional Sciences Martin Luther University Halle‐Wittenberg Halle (Saale) Germany; ^2^ Faculty of Agriculture/Environment/Chemistry HTW Dresden – University of Applied Sciences Dresden Germany; ^3^ Department of Clinical Veterinary Medicine, Vetsuisse Faculty, Clinic for Ruminants University of Bern Bern Switzerland; ^4^ Vetsuisse Faculty, Institute of Genetics University of Bern Bern Switzerland; ^5^ Saxon State Laboratory for Health and Veterinary Affairs Leipzig Germany

**Keywords:** aneuploidy, cattle, chromosomal abnormalities, disorder of sex development, malformation, precision medicine, urethra

## Abstract

Hypospadias occurs sporadically in male livestock and is characterized by a non‐fused urethra during fetal development. In this study, perineal hypospadias, a bifid scrotum, penile hypoplasia, and bilateral abdominal cryptorchidism were diagnosed in a neonatal Holstein male calf. Septicemia was also suspected due to hypothermia, blurred conjunctivae, and loss of sucking and swallowing reflexes. Gross pathology revealed that both testicles were located intraabdominally caudally to the kidneys. Histopathological examination of the hypospadias showed a urothelium‐lined mucosal fold and parts of the corpus cavernosum penis and prepuce in the subcutis. Whole genome sequencing was performed on the affected calf. Analysis of short‐read coverage depth along the chromosomes identified an entire extra copy of chromosome 26. Based on the comparison of available variant calling data from the sire, the identified trisomy 26 is due to non‐disjunction of homologous chromosomes during the generation of paternal gametes. We have shown for the first time an association between bovine hypospadias and trisomy 26, which adds to the understanding of variation in fetal male sexual development.

Disorders of sexual development (DSDs), such as hypospadias, are rarely reported in animals although, depending on their severity, they can be a significant reproductive and health problem. Hypospadias is a congenital abnormality of the male mammalian genital region characterized by a non‐fused urethra during fetal development (Fukami et al., 2006; Iannuzzi et al., 2021). The shared phenotype results from either a partial or complete failure in the formation or fusion of both urethral folds during phallus elongation (Hynes & Fraher, 2004; Kluth et al., 1988). This failure leads to the persistence of the urethral groove, which normally closes to form the tubular extrapelvic urethra (Smith et al., 2006). The fusion process typically begins in the perineal region and progresses towards the glans penis (Yamada et al., 2003); however, in cases of failure, it results in the absence of the median raphe in various parts of the genitalia, including the perineum, scrotum, penis (resulting in penile aplasia or hypoplasia), and prepuce (Alam et al., 2005; Switonski et al., 2012). Hypospadias is categorized into different types according to the location of the urethral opening: balanitic/glandular, penile, scrotal, perineal, and anal (McFarland, 1958; Smith et al., 2006).

Hypospadias has previously been described in several domestic animals including cattle, buffaloes, sheep, and goats as developmental abnormality in which the urethra of males opens on the underside of the penis or on the perineum (OMIA: 001187). Recently, a case series of bovine hypospadias was reviewed, including 39 males and two cases of female pseudohermaphroditism. In addition to hypospadias, other congenital anomalies were found including atresia ani and/or recti, imperforate ears, anorectal and cardiovascular anomalies, cryptorchidism, or renal and ureteral malformations (Iannuzzi et al., 2020). To date, no monogenic cause of hypospadias has been described in animals, although a single case report in cattle suggested chromosomal abnormalities as a possible cause (reviewed by Iannuzzi et al., 2020).

In humans, hypospadias is an important congenital developmental abnormality of the penis, which is frequently associated with mental retardation and cryptorchidism (Goldblatt et al., 1987; Khuri et al., 1981; Nassar et al., 2007). It is considered to be the second most common human DSD after cryptorchidism with an incidence of 1/330 in male births (Fredell et al., 2002) and is considered to be a complex disorder with both monogenic and environmental factors involved in its pathogenesis (OMIM: 300633).

The aim of our study was to characterize the clinicopathological phenotype of a male German Holstein calf with congenital hypospadias and to analyze whole genome sequencing (WGS) data to improve the understanding of this congenital anomaly.

This study did not require official or institutional ethical approval as it was not experimental, but rather part of clinical and pathological veterinary diagnostics. The calf in this study was examined by a veterinarian with the consent of the owner and handled according to good ethical standards.

A purebred German Holstein calf was delivered after a gestation period of 287 days (reference in Holstein heifers: 277.8 days) (Norman et al., 2009) on a dairy farm from a first calving of the dam at age 25.7 months at calving. The birth weight of the calf was 27.8 kg (reference in Holstein calves: 42 kg) (Korst et al., 2017). There was no history of previous disease or medical treatment of the dam. The sire was an artificial insemination bull also with no history of disease. The herd was free of brucellosis, leucosis, bovine herpesvirus 1, bovine viral diarrhea virus, and *Mycobacterium avium* ssp. *Paratuberculosis* infections.

The calf was presented to the farm veterinarian at age 2 days due to apathy and anorexia. On clinical examination at the farm, a 17 cm long mucosal fold was observed in the region of the penis, starting 5 cm ventral to the anus, with a urethral orifice above the scrotum. The scrotum was medially divided into two parts. A hypoplastic penis with a diameter of about 3 mm was found in the subcutis. The clinical findings were compatible with hypospadias (Figure 1a). Septicemia was also suspected due to hypothermia (rectal body temperature of 37.6°C), blurred conjunctivae and loss of the sucking and swallowing reflexes. Hypospadias in combination with other genital malformations such as bifurcated scrotum and abdominal cryptorchidism seems to be common (Iannuzzi et al., 2020). It cannot be excluded that the septicemia observed in this calf was due to an ascending urinary tract infection, as it has been reported in another calf with hypospadias (Park et al., 2012). Due to the poor prognosis, the calf was euthanized (pentobarbital sodium, 80 mg/kg i.v.; Euthadorm 500 mg/mL; CP‐Pharma, Burgdorf, Germany).

**FIGURE 1:**
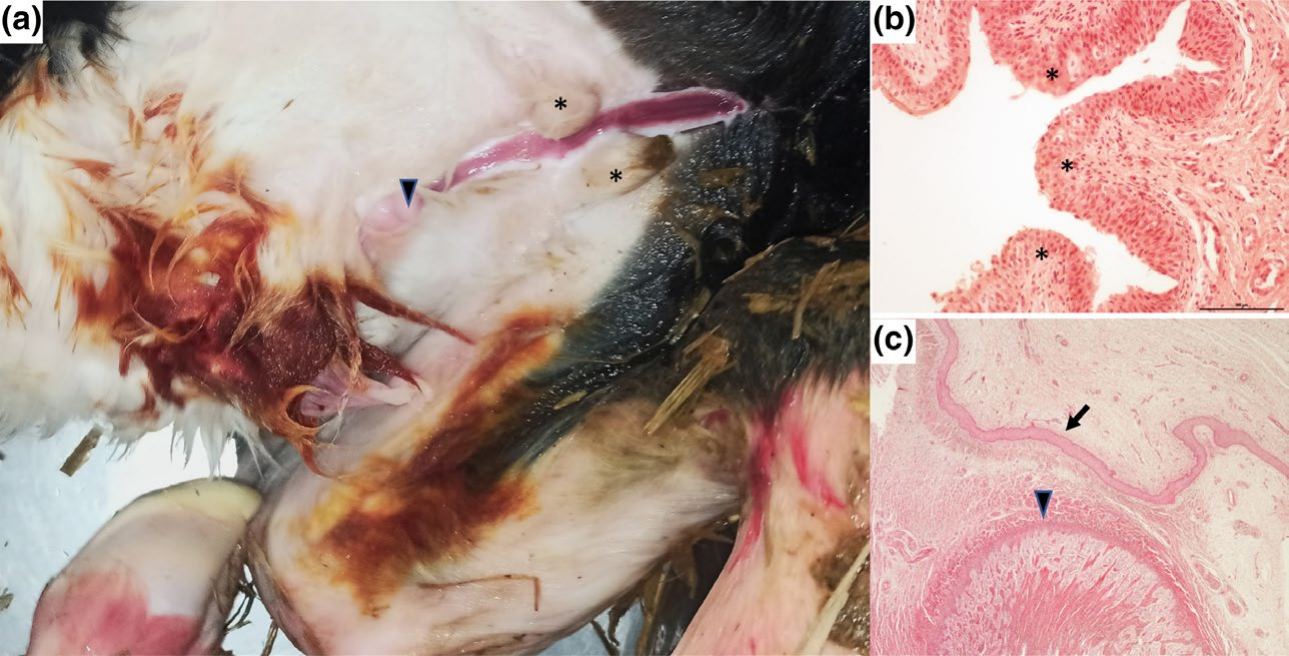
Hypospadias in a male Holstein calf. (a) Perineal hypospadias with urethral opening (arrow) 5 cm ventrally to the anus. An opened sagittal slit is running through a divided scrotum (asterisks) and ending at the prepuce with a hypoplastic penis (arrowhead). (b) Urothelial tissue (asterisks) as the inner lining of the open urethra. (c) Preputial epithelium (arrow) and corpus cavernosum of the hypoplastic penis (arrowhead). Hematoxylin and eosin staining, 20×.

Gross pathology revealed that both testicles were located intra‐abdominal and caudal to the kidneys. No evidence of the presence of Mullerian duct derivatives was observed. The lungs were marbled and only partially ventilated. The musculoskeletal system and internal organs of the calf were typical for its age and showed no abnormalities. Histopathological examination of the hypospadias showed a urothelium‐lined mucosal fold and parts of the corpus cavernosum penis and prepuce in the subcutis (Figure 1b,c). Bacteriological examination revealed moderate levels of ß‐hemolytic *Escherichia coli* in the liver, lungs, and kidneys, consistent with septicemia. Organ samples (lung, liver, kidney, and brain) were tested negative for *Coxiella burnetii* by PCR. The virological examination of the organ samples by virus cultivation in primary calf testicular cells and PCR (bovine viral diarrhea virus and Schmallenberg virus) was negative.

We hypothesized a possible genetic etiology for this disease and performed a genetic analysis. DNA was extracted from EDTA blood of the affected calf using standard methods and WGS was performed as previously described (Jacinto et al., 2021). Reads were mapped to the ARS‐UCD1.2 assembly (Rosen et al., 2020). Single‐nucleotide variants (SNVs) and small indel variants were called and an evaluation of possible larger structural variants was investigated as previously described (Jacinto et al., 2024).

Assuming a monogenic protein‐changing variant as the cause of this disorder, the WGS data were filtered and no homozygous but three heterozygous private variants with a predicted high or moderate impact effect on three different genes were identified (Table S1), but none of the variants affected a candidate gene for hypospadias. Subsequently, the presence of larger structural DNA variants and chromosomal abnormalities was examined by analyzing read depth or coverage along all 29 autosomes as well as the X and Y chromosomes revealed an entire extra copy of chromosome 26 (Figure 2a,b). The presence of single X and Y chromosomes were noted, confirming that the calf was indeed a XY male. Based on the comparison of available variant calling data from the sire, whose genome was previously sequenced for other reasons, the identified trisomy 26 appears to be due to non‐disjunction of homologous chromosomes during the generation of paternal gametes. Inspection of heterozygous SNVs along chromosome 26 showed that approximately two thirds of the variant‐containing reads were of clear paternal origin (Figure 2c).

**FIGURE 2:**
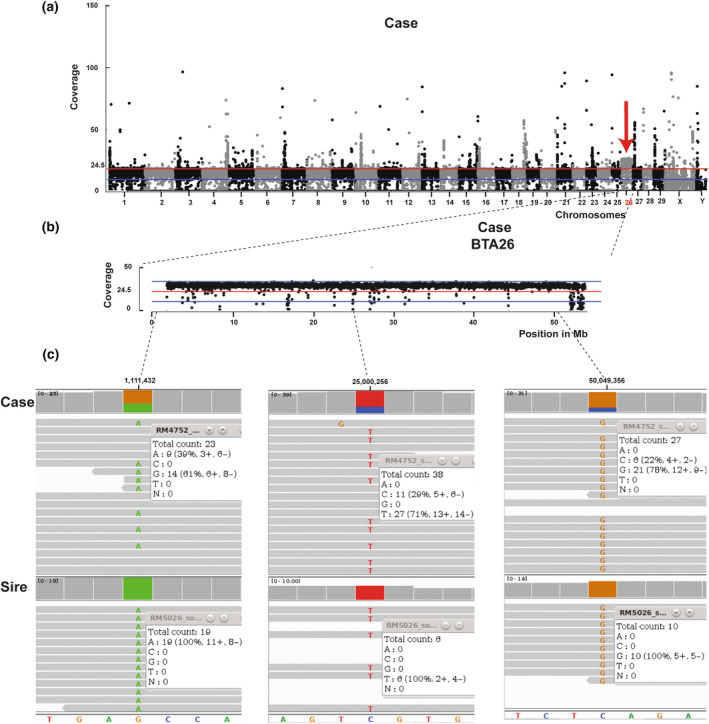
Trisomy 26 in a male Holstein calf with hypospadias. (a) Coverage plot of all 29 autosomes as well as the X and Y chromosomes with a 10‐kb window of the affected calf. Note the extra copy of chromosome 26. Red line: genome‐wide average coverage. (b) Coverage plot of chromosome 26 of the affected calf. Note the increasing of the coverage across all chromosomes 26 (trisomy). Red line: genome‐wide average coverage. (c) Integrative genomics viewer screenshots of three different regions of chromosome 26 in the calf and its sire. Note the three heterozygous SNVs in the calf showing that approximately two thirds of the variant‐containing reads are of clear paternal origin.

Genetic analysis of a case of bovine hypospadias has previously linked this form of DSD with a chromosomal abnormality, characterized by a non‐mosaic, pseudo‐monosomy 59, XY + tan(18;27) (Iannuzzi et al., 2020). This anomaly originated from a tandem fusion translocation involving bovine autosomes 18 and 27. This resulted in a loss of genomic sequences, mainly observed at the distal end of chromosome 18 and the proximal end of chromosome 27 (Iannuzzi et al., 2020). More recently, the simultaneous occurrence of hypospadias and bilateral lip and jaw cleft was reported in a Piedmontese × Wagyu crossbred calf (Marc et al., 2023). Genomic data analyses identified homozygous high impact SNVs affecting 13 different genes. The authors concluded that several genes were probably involved in the birth defects (Marc et al., 2023). However, none of these genes is located on chromosome 26, which was affected by trisomy in our case.

The morphogenesis of the genital tubercle involves various genes such as *BMP2*, *BMP4*, *BMP7*, *FGF*, *FGF10*, *FGF8*, *HOXA13*, *HOXA4*, *HOXB6*, *HOXD13*, *TGF*, and *WNT5A* (Chen et al., 2018, 2022; Murashima et al., 2015). Notably, the fibroblast growth factor 8 (*FGF8*) gene is located on bovine chromosome 26. Zimin et al. (2009) constructed a bovine–human synteny map that revealed three homologous synteny blocks on bovine chromosome 26 and human chromosome 10. Fine mapping analyses confirmed linkage of four chromosomal regions in human familial hypospadias, including a region on chromosome 10 (Söderhäll et al., 2015).

Aneuploidy is a genetic condition characterized by the loss or gain of one or more chromosomes and is rarely reported in cattle, probably often resulting in abortion (Schmutz et al., 1996). They can affect both the gonosomes and autosomes. Reported trisomies of entire bovine autosomes are rarely documented and primarily affect a limited number of chromosomes (i.e., 18, 20, 21, 22, 28, 26, and 29). These abnormalities are associated with conditions such as brachygnathia, blindness, retarded growth, organ hypo‐ or aplasia, and other malformations. While many of these chromosomal aberrations result in severe physical deformities and are lethal, a few cases have been observed in live born animals (Agerholm & Christensen, 1993; Christensen & Juul, 1999; Ducos et al., 2000; Gallagher et al., 1999; Häfliger, Agerholm, & Drögemüller, 2020; Häfliger, Seefried, & Drögemüller, 2020; Herzog et al., 1977; Iannuzzi et al., 2004; Lioi et al., 1995; Mayr et al., 1985; Schmutz et al., 1987, 1996).

To date, only one case of trisomy 26 has been reported in cattle. Using cytogenetic analyses, Ducos et al. (2000) found a trisomy 26 mosaicism in an infertile and growth retarded female Holstein–Friesian heifer. Histopathological analysis of the ovaries revealed a non‐functional character of the ovarian tissue characterized by total absence of functional ovarian tissue replaced by a fibroblastic proliferation associated with a mature collagen matrix. However, hypospadias in male cattle has never been described before in association with a trisomy 26 or other trisomies. In addition, trisomy of human chromosome 10, which is the homologous chromosome to bovine chromosome 26 (Zimin et al., 2009), is very rarely diagnosed in live‐born children. Case reports describe live births with human trisomy 10 mosaicism with typical clinical features including feeding problems, growth retardation, failure to thrive, blepharophimosis, low set ears, high arched palate, retrognathia, long slender trunk, marked plantar and/or palmar furrows, cardiopathy, and early death (Gao et al., 2018), but no DSD.

Although performing cytogenetics analyses such as G‐banding karyotyping or fluorescence in situ hybridization was not strictly necessary to validate our findings, we address the limitation that the applied WGS approach did not exclude the possibility that the affected calf had a mosaic karyotype with the presence of trisomic and normal cell lines.

In conclusion, we postulate that the detected extra copy of the entire chromosome 26 probably caused the observed hypospadias in the presented case. We have demonstrated for the first time an association between bovine hypospadias and trisomy 26, which expands the understanding of possible pathways for the development of this entity. WGS is a valuable method and a good alternative to karyotyping for the diagnosis of chromosomal abnormalities. Further research is needed to determine the formal pathogenesis of hypospadias or other forms of DSD in bovine cases of aneuploidy.

## AUTHOR CONTRIBUTIONS

**Markus Freick:** Conceptualization; formal analysis; investigation; resources; supervision; validation; visualization; writing – original draft; writing – review and editing. **Joana G. P. Jacinto:** Conceptualization; formal analysis; investigation; methodology; validation; visualization; writing – original draft; writing – review and editing. **Irene M. Häfliger:** Formal analysis; methodology; software; writing – review and editing. **Jim Weber:** Formal analysis; investigation; writing – review and editing. **Holger Behn:** Formal analysis; investigation; writing – review and editing. **Ruben Schreiter:** Formal analysis; investigation; writing – review and editing. **Cord Drögemüller:** Conceptualization; resources; supervision; writing – original draft; writing – review and editing.

## CONFLICT OF INTEREST STATEMENT

The authors declare no conflict of interest.

## Supporting information


Table S1.

## Data Availability

The WGS data are available under the study accession no. PRJEB18113 at the European Nucleotide Archive (www.ebi.ac.uk/ena; case: SAMEA112689389, sire: SAMEA114347186).
